# Bio-Pesticides: New Tool for the Control of *Aedes* (*Stegomyia*) *albopictus* (Culicidae: Diptera) in Pakistan

**Published:** 2017-05-27

**Authors:** Hazrat Bilal, Sumrin Sahar, Sadrud Din

**Affiliations:** 1 Medical Entomology and Disease Vector Control, Health Services Academy, Islamabad, Pakistan; 2 University of Copenhagen, Copenhagen, Denmark; 3 Water, Agriculture and Technology Transfer Program, Kabul, Afghanistan

**Keywords:** Mosquitoes, Plant extracts, Larvicide

## Abstract

**Background:**

Application of plant extracts as mosquito control strategy was practiced from centuries. These are easily available, non-toxic, biodegradable and exhibit broad-spectrum target specific activities against larval stages of mosquitoes.

**Methods:**

Different potential parts of locally grown plants, seeds of nutmeg (*Myristica fragrans*), peel of musambi (*Citrus sinensis*), leaves of babuna (*Matricaria chamomilla*)*,* mint (*Mentha spicata*) and ginger rhizome (*Zingiber officinale*) selected and evaluated for their larvicidal properties against *Aedes* (*Stegomyis*) *albopictus*. Oils were extracted through steam distillation process and extracts were evaluated as per WHO 2005 guidelines for testing of insecticides against larvae of mosquitoes.

**Results:**

Among the five plant extracts, *C. sinensis* had the lowest LC_50_ (400.81ppm) while *M. fragrans* had the highest LC_50_ value (710.30ppm) respectively after 24h of exposure. In terms of % age mortality, a series of concentrations (300–800ppm) gave high % mortality in case of *C. sinensis* while *M. fragrans* gave low % age mortality.

**Conclusion:**

All the five plant species have larvicidal effects to certain extant and *C. sinensis* had great potential. Further small-scale field trials with the extracts of the most promising one (*C. sinensis*) shall be conducted to determine operational feasibility.

## Introduction

Dengue, malaria, filariasis, yellow fever and Japanese encephalitis are the most important diseases transmitted by mosquitoes (Rozendaal 1997). Fifty million cases of dengue occur globally every year (WHO 2009). Dengue has now emerged in many countries, especially in Pakistan where occurred outbreaks affected the socio-economic development in the Region (Savioli and Velayudhan 2014). Dengue epidemic in Pakistan (2011) is being observed where, more than 22,778 cases confirmed and 353 deaths reported (Anonymous 2011). In 2013, a Punjab and Khyber-Pakhtunkhwa (KPK) provinces hit by second epidemic, in northern areas of Pakistan the human mobilization from dengue endemic regions, geographic expansion of dengue fever vector due to importation, climatic change, all are the factors, which resulted in the emergence of dengue in northern areas (Ali 2013). In district Swat (KPK) 6,000 dengue cases with 47 deaths were reported (Khan and Khan 2013).

Worldwide mosquito control depends on the application of synthetic insecticides as a part of Integrated Vector Control (IVM) Programmes (Becker et al. 2010). Toxic effects and resistance to synthetic insecticides are barriers in controlling mosquitoes. Therefore, it is necessary to develop safe alternative insecticides, which required minimum care (Mittal and Subbarao 2003).

Plant based insecticides may be the best option for mosquito control as they have biologically active chemicals that are easily decomposed into products which are not toxic to other species (Sanjay and Tiku 2009) and potentially suitable for use in control of mosquito larvae (Yang et al. 2004).

In fact, many researchers have reported the effectiveness of plant extracts or essential oils against mosquito larvae (Rahuman et al. 2008). Research on insecticidal properties of botanicals inferred that they are bio-degradable, environmentally safe and target specific (Govindarajan et al. 2008). (Muthukrishnan 2012) and (Bilal et al. 2012) evaluated the larvicidal effects of extracts from *Cinnamomum cassia*, *Citrus sinensis* Linnaeus var musambi, *Tribulus terrestris*, *Eucalyptus camaldulensis*, *Piper nigera*, *Ricinus communis*, *Allium sativum*, *Linum usitatissimum* and *Citrus sinensis* L var succari against *Aedes albopictus*. A number of other researchers which have used plant products for the mosquito control like (Komalamisra et al. 2005) reported the ether extracts of *Trigonostemon reidioides*, *Rhinacanthus nasutus*, *Derris elliptica*, *Homalomena aromatica*, *Stemona tuberose*, *Acorus calamus* and *Piper nigrum* (Siddiqui et al. 2004), *Artemisia annua*, *Sonchus oleraceus* and *Chenopodium album* (Sharma et al. 2006) *Solanum xanthocarpum* (Mohan et al. 2005) *Argemone mexicana* (Sakthivadivel and Thilagavathy 2003).

In the view of increased interest in development of plant-based insecticides as an alternative to synthetic insecticide, this study was planned and conducted to assess the larvicidal potential of five medicinal plants against the medically important mosquito (*Aedes* (*Stegomyia*) *albopictus*).

## Materials and Methods

### Collection of Plants

Nutmeg (*M. fragrans* Houtt) seeds, musambi (*C. sinensis* (L) Osbeck) peel, babuna (*M. chamomilla* L) leaves, Mint (*M. spicata* L) leaves and ginger (*Z. officinale* Roscoe) rhizome were collected from botanical garden of University of Agriculture Faisalabad (31.4339° N, 73.0649° E) and local market of Faisalabad (31.4181° N, 73.0776° E).

### Extraction of oil

The seeds, peel, leaves and rhizomes were washed, then dried and later pulverized in an electric grinder (Anex Germany). The pulverized material was placed in thimble and kept in extraction tube of Soxhelt apparatus with extractor ID 38mm, extractor volume 85ml and flask volume 250ml (Vogel 1978) for the extraction of oil by steam distillation method using ether as solvent (250ml/20g sample). The cycle time for one sample was 4– 5h. Solvent was evaporated at room tempeature, leaving oil, collected in flask. Stock solution was prepared by adding 1ml of oil from each plant in 99ml of ether and considered as 1% stock solution from which series of concentrations (%) were prepared (Akram et al. 2010).

### Collection and Rearing of Mosquitoes

Mosquito larvae were collected from potential breeding sites of *Aedes* around Islamabad (33.7167° N, 73.0667° E) with a standard pipette while adults were collected by battery-operated aspirator. Larvae were reared for mass population in the insectary of Medical Entomology and Disease Vector Control department, Health Services Academy, Islamabad. The first instar larvae were fed with fat free milk powder while other instars larvae were fed with Tetra-Min fish feed powder at 28±2 °C and 75±5% humidity. Adults were reared in screened cages by providing 10% sucrose solution while female mosquitoes were also fed on the blood of albino rats (Imam et al. 2014). Larvae of *Ae.* (*Stg.*) *albopictus* were identified using identification key of Leopoldo (Leopoldo 2004).

### Larvicidal Bioassay

The extracted oils were used in six different concentrations along control with three replicates for each treatment, each replicate containing 200ml of the oil solution in 250 ml Pyrex glass beakers. A batch of fifteen 3^rd^ instar larvae of the *Ae.* (*Stg*.) *albopictus* were exposed in each beaker containing oil solution (WHO 2005), while control was treated with ether only. Mortality of larvae was counted after 24 hours. The experiment performed under lab conditions at 28±2 °C and 75±5% relative humidity.

### Statistical Analysis

Abbot’s formula (Capinera 2008) was used for correction of mortality and the data so obtained was analyzed by probit analysis (WHO 2005) by using MANITAB-15 software for dose mortality regression line and % age mortality graph were prepared using Microsoft office 2007.

## Results

The crude ether extracts of nutmeg (*M. fragrans*) seeds, musambi (*C. sinensis*) peel, babuna (*M. chamomilla*) leaves, mint (*M. spicata*) leaves and ginger (*Z. officinale*) rhizome had been evaluated as potential source of insecticides. Results on the larvicidal activities of extracts were reported in the present study (Table 1) confirms their potential for the control of *Ae.* (*Stg.*) *albopictus* larval population. All extracts showed moderate larvicidal effects however, the highest larval mortality was found in musambi peel with 400.81ppm LC_50_ value, followed by babuna (438.60ppm), ginger (502.55ppm) while mint and nutmeg had the highest LC_50_ value (596.94ppm and 710.30ppm) respectively after 24h of exposure.

**Table 1.** Larvicidal activity different plant extracts at different concentrations against 3^rd^ instar larvae *of Aedes* (*Stg*.) *albopictus*

**Plants**	**Con.**	**% mortali**	*LC_50_ (ppm)	**95% FL (LFL–UFL)**	**2**	**p**
**Musambi** ***C. sinensis***	300	32	400.81	359.82–435.22	4.62	0.32
	400	46				
	500	54				
	600	61				
	700	70				
	800	82				
**Nutmeg** ***M. fragrans***	300	10	710.30	654.35–793.80	4.60	0.33
	400	16				
	500	25				
	600	29				
	700	43				
	800	58				
**Babuna** ***M. chamomilla***	300	27	438.60	394.18–477.52	0.18	0.99
	400	41				
	500	51				
	600	61				
	700	66				
	800	70				
**Mint** ***M. spicata***	300	31	596.94	521.21–719.96	1.11	0.89
	400	38				
	500	42				
	600	47				
	700	55				
	800	62				
**Ginger** ***Z. officinale***	300	35	502.55	437.55–570.33	1.05	0.90
	400	39				
	500	47				
	600	56				
	700	61				
	800	67				

* LC_50_ ie, lethal concentration (%age) to kill 50% population of the subjected organism

**Fig. 1.**
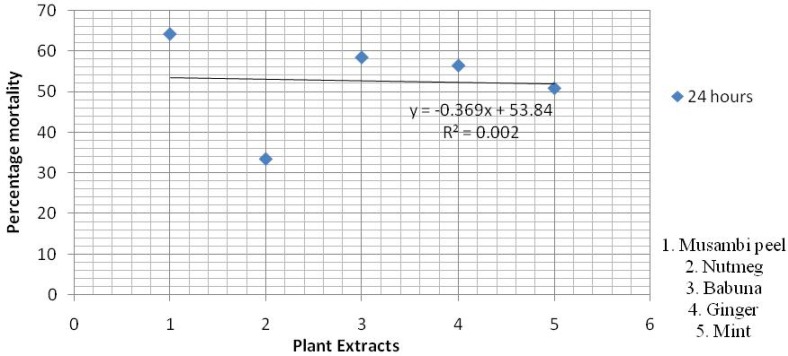
Percentage mortality of different plant extracts against *Aedes* (*Stg*.) *albopictus* larvae after 24 hours of exposure

In terms of % mortality musambi had the high mortality (64.25%) followed by babuna and ginger (58.51 and 56.48%) respectively, while nutmeg and mint had the lowest % mortality (33.51 and 50.92%) respectively after 24h of exposure as shown in Fig. 1. The percentage of mortality was directly proportional to concentration of the extract (Table 1). After exposure to the test concentrations, the treated larvae exhibited restlessness, tremors, sluggishness and convulsions followed by paralysis at the bottom of the bowl.

## Discussion

Recently *Ae.* (*Stg.*) *aegypti* along with *Ae.* (*Stg.*) *albopictus* played havoc in different parts of Pakistan. Different control measures have been adopted with the major focus on chemical control. Resultantly, occurrence of insecticide resistance in mosquitoes and other public health pests have been reported (Khan et al. 2011, 2013). Their residues in the environment and effects on humans and non-target organism are major problems due to which investigators are now directing their attentions towards the development of plant based insecticides (Biopesticides).

Various compounds, including phenolics, terpenoids, and alkaloids, exist in plants (Wink 1993, Swin 1977, Kim et al. 2001) and may jointly or independently contribute to the generation of larvicidal activities of mosquito (Assabugi et al. 1997, Hostettmann and Potterat 1997).

Outcome of five different plant oils, which were, used against the 3^rd^ instar larvae of *Ae.* (*Stg.*) *albopictus* are shown in Table 1. Results were satisfactory and showed efficacy. Larval mortality increased with increase in dose of plant oil and at higher doses, it gave more than 80% mortality without any pupal or adult emergence. While in control, there was less than 5% mortality after 24h. *Citrus sinensis* peel extract gave reasonably good results (400.81ppm) against the larvae of *Ae.* (*Stg.*) *albopictus* when compared with other tested plant oils, like findings of Warikoo et al. (2012) they concluded that *C. sinensis* leaf extracts had 446.84ppm LC_50_ value against the larvae of *Aedes aegypti as* Citrus plants contain limonoids which work both as toxicant and feeding deterrents (Akram et al. 2010) and also has insecticidal effects (Bilal et al. 2012). Thus, the larvicidal activity of *Citrus sinensis* is due to limonoids, in addition to alkaloids, saponins, steroids, flavonoids and tannins. While on the other side, previous workers (Michaelakis et al. 2009, Din et al. 2011) reported LC_50_ values for some citrus peel and seed oils against the larvae of *Ae. albopictus* and *Cx. pipiens* different than the obtained values in the present investigation.

In our findings, *M. fragrans* had least effectiveness and gave 50% mortality after 24h of exposure is not in agreement with the studies of Senthilkumar et al. (2009). They tested *M. fragrans, Eucalyptus globulus*, *Artemisia annua*, *Cymbopogan citratus*, *Justicia gendarussa*, *Annona squamosa* and *Centella asiatica* and found that all gave 80–100% mortality against larvae of *Anopheles stephensi.* It is well documented that toxicity values of a substance may be largely varied due to several factors (Busvine 1971) attributed to the test conditions (temperature, light, humidity, exposure period and solvent); tested species (age, stage and susceptibility) and tested plant material (season, location, extraction method and used part) and there may be different constituents in a botanical extract may interact with each other’s, leading to synergistic or antagonistic effects (Mansour et al. 2000, 2003).

## Conclusion

Out of the 5 plants extracts, *C. sinensis* has good larvicidal potential against larvae of *Ae.* (*Stg.*) *albopictus* in terms of LC_50_ and % age mortality. Therefore, we suggest that *C. sinensis* extracts as well as other plant extracts should be investigated and compared with other plant extracts, which already been tested against mosquitoes.
